# Therapeutics

**Published:** 1879-08

**Authors:** 


### THERAPEUTICS.

SYPHILIS IN RELATION TO MARRIAGE.—(*Gaz. des Hôpitaux*).

The following are, in brief, the points made by Dr. Fournier in two lectures delivered in January:

A man may marry after having had syphilis, but he ought to marry only on certain conditions, which we must determine. One who marries with unextinguished syphilis is dangerous as a husband, father and a member of society.

1. He is dangerous as a husband, by reason of lesions to which his wife is exposed; and in two ways. One, by simple, vulgar contagion, in the intimate relations of conjugal life; the other, by placental contagion, the mother, being or having been pregnant, having contracted the disease from the infant, and not directly from the husband. Contagion through placental exchange is an absolute fact.

2. He is dangerous as a father. It should be held as a constant fact that a syphilitic father is a source of danger to the offspring; nevertheless, sypilitic heredity proceeding from the father alone, the mother remaining unaffected, is much less terrible than has been asserted. For an infant born of syphilitic parents there are three alternatives. (a) Either it is dead at birth, or (b) it will be born at term subject to the disease, or (c) it will survive with compromised health.

3. He is dangerous as a member of society. This consideration is too self-evident, and its moral and physical bearings too generally admitted to require reproduction. Let it be sufficient to call attention to the immense responsibility to society he incurs by marrying when thus infected.

Passing now to consider the conditions under which a syphilitic should be allowed to venture on matrimony, we find that they enumerate themselves as follows:

1. The absolute absence of actual symptoms. This cause of exclusion seems so obvious that it seems superfluous to insist upon it; yet we must not be deceived, for examples of this incredible and criminal audacity have been observed over and over again.

2. The advanced period of the disease. We may commit to memory this axiom—the younger the syphilis of the husband, the graver are the dangers he conveys by marriage; and conversely, the older the disease the more favorable the chances of escape. It is recent syphilis which causes most danger by contagiousness, and it is in the first six, ten or twelve months that its manifestations are more disseminated, dangerous and disposed to relapse. Nothing relapses like a condyloma, and we may see erosions of the mouth reproduced in smokers ten or twenty times within a short period. The recent accidents of syphilis are so much the more redoubtable, in that their favorite sites are the mouth and genitals—localities especially dangerous in relation to contagion in married life. In the secondary stage, also, the phenomena are more dangerous only because apparently so benign and insignificant. In their apparent benignity is their greatest danger. The conclusion to be drawn is, that the advanced age cf a syphilis is an essential condition for marrying. What should be its duration? This is an extremely delicate point, but in order to lay down a rule, we may say that a syphilitic should not dream of marrying until a minimum of four years has passed; and he had better wait still longer.

3. A certain period of immunity should have elapsed since the last accident. This offers a guaranty which is, for several reasons, necessary. For this period of repose we should fix a minimum limit of eighteen months to two years.

4. The unmenacing character of the syphilitic diathesis. There assuredly exist slight and grave syphilis; the former confined to a small number of superficial accidents, while the latter give rise to deep seated lesions, even when treated. Now the quality is a very essential condition to be considerd. The three principal types of the latter, or grave form, are: (a) That one remarkable for the incessant reproductions of the same accidents, though these may be very superficial. (b) Those which are grave on account of intensity, multiplicity and nature of the accidents produced, and which at an early period affect the tertiary form, become visceral, and remain refractory to treatment. (c) That which invades organs essential to life, e. g., the brain, cerebral syphilis being especially dangerous.

5. A sufficiently prolonged specific treatment. This is the capital condition, par excellence. We may at the present time affirm that syphilis treated energetically at its commencement, and for a sufficiently long time, has no tertiary period. Treatment also diminishes and suppresses the cause of contagion and chances of heredity.

Conclusions. To any syphilitic subject who does not fulfil the above five indispensable conditions, we ought to refuse marriage. Rarely will an imprudent person, who has married in spite of the advice of his physician, not have to repent it, while those who have married with the consent of their medical adviser have neither proved dangerous to their wives nor children.—*Med. Times and Gazette.*

